# Effect of the herbal mixture composed of *Aloe Vera*, *Henna*, *Adiantum capillus-veneris*, and *Myrrha* on wound healing in streptozotocin-induced diabetic rats

**DOI:** 10.1186/s12906-016-1359-7

**Published:** 2016-10-06

**Authors:** Hamid Galehdari, Samira Negahdari, Mahnaz Kesmati, Anahita Rezaie, Gholamreza Shariati

**Affiliations:** 1 Toxicology Research Center, Ahvaz Jundishapur University of Medical Sciences, Ahvaz, Iran; 2 Department of Genetics, Shahid Chamran University, Ahvaz, Iran; 3 Department of Pathobiology, Faculty of Veterinary Medicine, Shahid Chamran University of Ahvaz, Ahvaz, Iran; 4 Department of Medical Genetics, Faculty of Medicine, Jundishapur University of Medical Sciences, Ahvaz, Iran

**Keywords:** Wound healing, Diabetic, Matrix metalloproteases, Herbal remedies

## Abstract

**Background:**

Wound healing is often impaired in diabetic animals and humans. Matrix metalloproteases act as pro-inflammatory agents in physiological wound healing pathways by stimulating cytokines including the interleukins, IL6, IL1A and IL1B, and the tumor necrosis factor and transforming growth factor beta1. Botanicals are traditionally used to assist healing of different types of wounds, because they produce fewer side effects. Our specific aim here was to develop a plant-based recipe supporting effective wound healing in diabetic animals.

**Methods:**

Plant materials from *Adiantum capillus-veneris*, *Commiphora molmol*, *Aloe Vera*, and *henna* were collected for this study, and oven-dried at 60 °C. The dried leaves and resins were then crumbled into a powder and mixed in equal parts with Vaseline as a preservative*.* This mixture was used as an ointment on wounds induced in 60 diabetic and non-diabetic rats that were divided into 6 subgroups receiving agent or control treatments. Necrotic tissue surrounding the wound was periodically removed during wound healing. RNA was extracted from the healing region of the wound at days 7, 14 and 21 for cDNA synthesis to monitor changes in *Tgfb1*, *Mmp3*, *Mmp9*, *Il6* and *Tnf* α expression using real-time PCR.

**Results:**

The expression of the *Mmp3*, the *Tnf* α, and the *Tgfb1* genes from wound tissue were significantly different (*p* < 0.05) between diabetic and non-diabetic (control) rats treated with the herbal mixture after 14 and 21 days. There was no significant difference (*p* > 0.05) of the *Mmp9* gene expression in diabetic and non-diabetic rats treated only with Vaseline after 7, 14, and 21 days. But, the expression of the *Mmp9* gene decreased significantly (*p* < 0.05) in diabetic rats after 14 days in comparison to non-diabetic rats, when the herbal mixture was added to Vaseline.

**Conclusions:**

Our study presents an herbal treatment that alters the gene expression signature at wounds induced in the rat model for type I diabetes in a manner consistent with accelerated healing, and demonstrates that this herbal treatment might be effective to treat wounds in diabetic patients.

## Background

Wound healing in individuals suffering diabetes requires the coordination of several cellular processes [1]. Incurable wounds in diabetics are mostly linked to postponed cellular penetration, formation of granulation tissue, diminished collagen, reduced blood circulation, increased blood viscosity and reduced angiogenesis [2]. The observation of impaired wound healing in diabetic humans has been mimicked in animal models such as streptozotocin-induced diabetic rats [3, 4]. The destruction of the extracellular matrix and its modification by matrix metalloproteases is a feature of leukocyte invasion, angiogenesis, re-epithelialization and tissue repair [5–7]. Restricting protease production is crucial to regular wound healing since maintaining high levels of specific matrix metalloproteases can degrade the matrix and compromise repair [8, 9]. Matrix metalloproteases can also stimulate cytokines and chemokines and promote pro-inflammatory activity for wound healing. This indicates an association between diabetes-stimulated inflammation and matrix metalloprotease expression [10–12]. Elevated levels of matrix metalloproteases have been reported in hyperglycemic human and mouse models [2, 13]. Many cytokines and growth factors are involved in wound healing. Studies have shown that pro-inflammatory cytokines in human and animal diabetic models, such as interleukins, IL6, IL1A, IL1B, and the tumor necrosis factor (TNF), become elevated immediately after wounding [14–16]. TGFB1 is a multifunctional cytokine and increases formation of granulation tissue and collagen synthesis during wound repair [17, 18].

Current wound treatment methods, including wound debridement, irrigation or grafting and wound treatment with antibiotics or proteolytic enzymes can have side effects [19]. However, herbal-based drugs with curative value for various disorders have been described in traditional medicine. Numerous drugs derived from plant resources improve healing of different types of wounds [20, 21]. Among plants with wound healing capacity, *Aloe Vera* (*Liliaceae*) with known anti-fungal, anti-microbial, anti-diabetic, and hypoglycemic properties has been used in traditional medicine as a cathartic or remedy for burns and wounds [22, 23]. Morgan et al. reported that *Aloe Vera* initiates angiogenesis and wound repair by up-regulating *VegfA* and the *Tgfb1*expression [24]. Vija Yalakshmi et al. reported that *Aloe Vera* acts as an anti-inflammatory agent by inhibiting *MMP9* expression in peripheral mononuclear blood cells [25]. Studies have indicated that the use of oral or topical *Aloe Vera* affects phases of inflammation, collagen synthesis, maturation and wound closure to improve wound healing in the diabetic rat model [26–28]. *Commiphora molmol* (Myrrha) produces resin with anti-bacterial, anti-fungal and anti-diabetic properties [29, 30], and is currently used to treat wounds, intestinal disorders, diarrhea, coughs and inflammation [31, 32]. Terpenoids, steroids, flavonoids, sugars and lignums are all present in *Commiphora molmol* [33]. Lotfy et al. reported that the combined use of honey, bee propolis, and Myrrha promoted wound healing in a patient with diabetes mellitus [34]. *Adiantum capillus-veneris* also has a long history in traditional medicine and exhibits anti-inflammatory, anti-diabetic, anti-infective, anti-microbial, and anti-oxidant properties [35]. *Adiantum capillus-veneris* has significant angiogenic capacity of wound healing *in vitro* [36]. These properties suggest that local administration of *Adiantum capillus-veneris* could have healing capability. Henna (*Lawsonia inermis*) is a well-known plant that is widely used to treat headaches, boils and skin inflammation. Experimental and clinical studies have demonstrated that Henna has anti-bacterial and anti-fungal properties, and exhibits hypoglycemic and anti-hyperglycemic effects that assist wound healing [37, 38].

Here we investigated whether a combination of the botanicals, *Adiantum capillus-vernis*, *Commiphora molmol*, *Aloe Vera* and Henna, can improve the wound healing effectively in streptozotocin-induced diabetic and non-diabetic rats.

## Methods

### Collection and preparation of the herbal mixture

Fresh *Aloe Vera* leaves were collected from the botanical garden at Jundishapur Ahvaz University of Medical Sciences and their identity verified by the Department of Horticulture (No.93). Shoots of *Adiantum capillus-veneris* (*A. diantaceae*) were collected from the Lorestan province in Iran (no. 1661). Fresh henna leaves were collected in the city of Kerman, Iran (KF 1408). The oleo gum resin of *Commiphora mol* was obtained from Saudi Arabia. The origin of plant materials were systemically identified and approved in the herbarium of Shahid Chamran University of Ahvaz, Iran. After collecting plants, Henna and *Aloe Vera* fresh leaves and *Adiantum capillus-veneris* shoots, they were washed and oven-dried at 60 °C. The dried leaves and resin of the *Myrrha* were then crumbled in a blender to create a fine powder that was mixed in equal parts with Vaseline as a preservative.

### Animals and treatment groups

Sixty adult male Wistar rats (200–220 g) were used in this study, which was authorized by the Medical Ethics Committee of Ahvaz Jundishapur University of Medical Sciences.. Rats were housed separately in a temperature-controlled (22–24 °C) room on a12 h light/dark cycle, and food and water were provided *ad libidum*. Type I diabetes mellitus was induce in half the animals used for this study, then diabetic and non-diabetic and diabetic animals were further divided into 2 treatment subgroups (Table 1).

**Table 1:** Each group was classified into two subgroups including only Vaseline treatment and Vaseline plus herbal mixture. It is noted that because Vaseline was used in this study as preservative, we had have to show its putative effect on gene expression in comparison to plant mixture. Each of subgroups contain five rats (*n* = 5). The gene expression was measured in periods of 7, 14, and 21 days after treatment with Vaseline or Vaseline and mixture

Time (day)	Group I: non-diabetic		Group II: diabetic	
	Vaseline	Herbal mixture & Vaseline	Vaseline	Herbal mixture & Vaseline
7	5 rats	5 rats	5 rats	5 rats
14	5 rats	5 rats	5 rats	5 rats
21	5 rats	5 rats	5 rats	5 rats

### Induction of type 1 diabetes mellitus

After a fasting period of 12 h, diabetes was induced in the test rats by intra-peritoneal injection of 60 mg streptozotocin (Sigma-Aldrich; USA) in saline-sodium citrate buffer per kg body weight. The rats consumed 5 % glucose solution all night to neutralize the hypoglycemia stimulated by the streptozotocin. Seven days after injection of streptozotocin, the blood glucose concentration was measured in the rats using a glucometer. One week post-injection, type 1 diabetes was identified in each rat by a blood glucose level that was consistently above 250 mg/100 ml. Diabetic rats exhibited typical symptoms of diabetes mellitus including polyuria, polyphagia and weight loss. Blood glucose levels were tested every 3 days in each rat throughout the study duration.

### Wound model

**Fig. 1:**
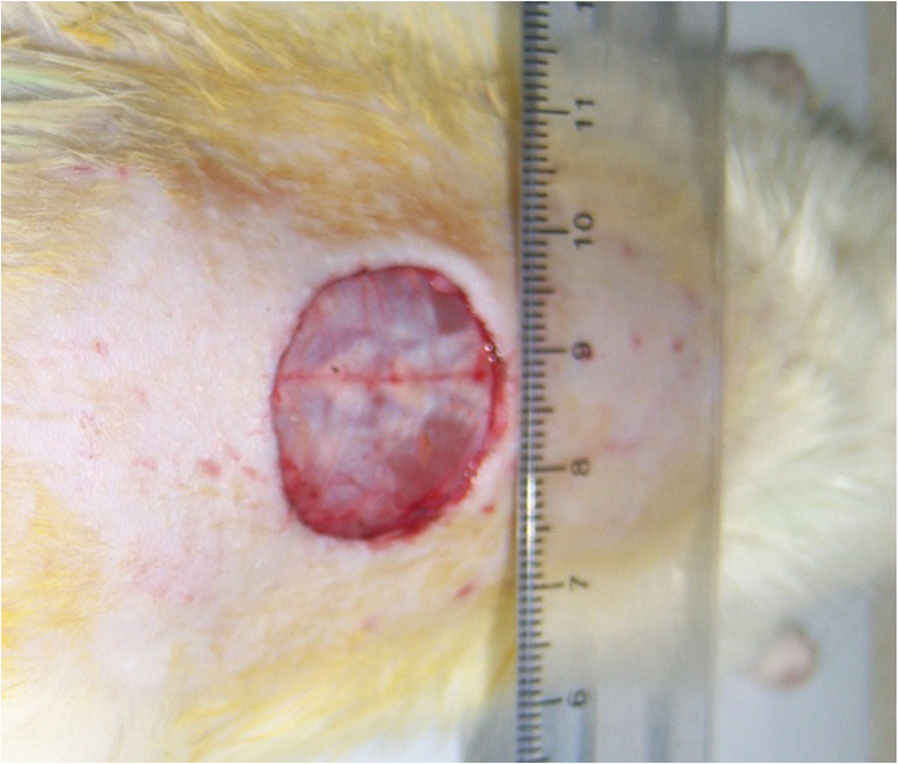
A 2-cm circular wound traversed both the epidermis and dermis on the back of each test rat at day 0

The wound was created on day zero on each rat in each treatment group. Rats were anesthetized by intra-peritoneal injection of ketamine (50 mg/kg) and xylazine (10 mg/kg). The fur on the back of each rat was first shaved off, and the wound site sterilized with 70 % alcohol before the skin wound was created by excising a circular region of skin about 2 cm in diameter (Fig.1).

### Tissue collection and expression analysis

Rats were euthanized with chloroform on days 7, 14, and 21 post-wounding. The wound and an edge of approximately 2 mm of adjacent skin were excised. The tissue sample was submerged in RNAlater (Qiagen; Germany) overnight at 4 °C. Total RNA was extracted from the tissues using the RNeasy Fibrous Tissue Mini Kit (Qiagen; Germany) according to manufacturer’s instructions, and stored at -80 °C. The RNA was reverse transcribed using the Quanti Tect Reverse Transcription Kit (Qiagen; Germany) as per instructions. The obtained cDNA was used for real-time PCR with master SYBR Green I (Takara Bio; Japan) on the ABI 7900HT. Real-time PCR was executed at 95 °C for 10 s, 62 °C for 15 s, and 72 °C for 8 s using primers for the normalizing gene, glyceraldehyde 3-phosphate dehydrogenase)*Gapdh*(and target genes, *Mmp3*, *Mmp9*, *Tgfb1*, *Il6*, and *Tnf*. Primers were designed by Gen Script according to the cDNA sequences of rat *Mmp3*, *Mmp9*, *Tgfb1*, *Il6*, *Tnf* and *Gapdh* in Gene Bank (Table 2). Real-time PCR was carried out twice on every cDNA sample. Target gene expression was monitored in wound tissue collected on days 7, 14 and 21 from diabetic and non-diabetic rats treated with either the herbal mixture in Vaseline as preservative or Vaseline alone.

**Table 2:** Sequence of designed primers for each gene is shown as forward and reverse. The primers used here for real time PCR were designed by www.GeneScript.com according their accession number

Gene	GenBank accession Number	Forward primer (5′–3′)	Reverse primer (5′–3′)
GAPDH	[NM_017008.3]	ATGACTCTACCCACGGCAAG	CTGGAAGATGGTGATGGGTT
MMP-9	[NM_031055]	TCGAAGGCGACCTCAAGTG	TTCGGTGTAGCTTTGGATCCA
MMP3	[NM_133523]	TCTTTCACTCAGCCAATGCT	GGGAGGTCCATAGAGGGATT
IL-6	[NM_012589.1]	AGTCCGGAGAGGAGACTTCA	TTGCCATTGCACAACTCTTT
TNFα	[NM_012675.3]	CCACCACGCTCTTCTGTCTA	GCTTGGTGGTTTGCTACGA
TGFβ1	[NM_031131.1]	CTGAACCAAGGAGACGGAAT	GGTTCATGTCATGGATGGTG

RNA isolated from the healing wound samples 7, 14 or 21 days after wound induction were reversed transcribed then gene expression analyzed in multiplex real-time PCR assays. Melting curves were obtained for each gene to validate data, and curves confirmed the accuracy of the peak corresponding to the gene of interest and strings of primer dimer. A standard curve was plotted to evaluate reaction efficiency using different dilutions of cDNA before real-time PCR multiplex assay implementation.

### Data analysis

Differences in gene expression were determined between treatment groups using the REST relative expression software tool (version 2009). REST calculates the relative expression of group means for target genes, which in our case were *Tgfb1*, *Mmp3*, *Mmp9*, *Il6* and *Tnf* α normalized for expression of the *gapdh* housekeeping gene in the treatment and control groups. Results were considered statistically significant when *p* values < 0.05.

## Results

We used both visual assessment and gene expression to assess the effect of an herbal mixture on healing of wounds induced in diabetic and non-diabetic rats.

The present investigation determined changes in the expression of the *Tgfb1*, *Mmp3*, *Mmp9*, *Il6* and *Tnf* α genes by real-time-PCR at the day 7, the day 14 and the day 21 post-wounding in the diabetic and non-diabetic rats. For data validation, melting curves were obtained for each gene. The curves confirm the accuracy of the peak corresponding to the gene of interest and strings of primer dimer. A standard curve was plotted to evaluate the efficiency of the reaction using different dilutions of the synthetic cDNA before implementation of real-time PCR.

**Fig. 2:**
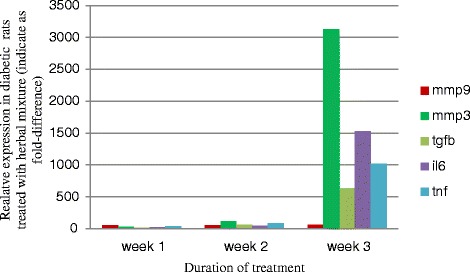
Fold-difference in gene expression in tissue removed from wounds in diabetic versus control rats treated with herbal mixture at 7, 14 and 21 days (indicated as week 1, week 2 and week 3) after treatment. The diagram signifies here the increased expression of mentioned genes in diabetic rats after 3 weeks treatment with herbal mixture

However, data showed significant difference (*p* < 0.05) in the expression of the *Mmp3*, the IL6, *Tnf* α and *Tgfb1*genes between diabetic and non-diabetic rats 14 daysays and 21 days after treatment with the herbal mixture at the wound site (Fig. 2). The *Mmp9* gene expression was firstly decreased at the day 14 and increased again at the 21th day in diabetic rats compared to non-diabetic rats treated with herbal mixture (*p* < 0.05) as has been shown in Fig. 2.

*Tgfb1*, *Mmp3*, *Il6* and *Tnf* α expression steadily increased during would healing, as shown by the significant increases between days 7 and 14 and days 14 and 21 in either diabetic or non-diabetic rats treated with the herbal mixture.

**Fig. 3:**
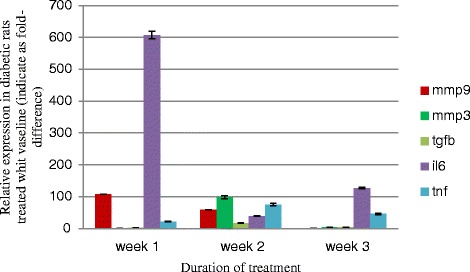
Fold-difference in gene expression in tissue removed from wounds induced in diabetic versus control rats treated with the Vaseline control at days 7, 14 and 21 of the wound healing process

The expression of *Mmp3* at 14 d, *Il6* at 14 and 21d, *Tnf* α at 21d and *Tgfb1* at 7, 14 and 21 days post-wounding in diabetic rats treated with Vaseline was significantly different (*p* < 0.05) from that of non-diabetic rats (Fig. 3). There was no significant difference (*p* > 0.05) for the *Mmp9* gene expression in diabetic rats and non-diabetic rats treated with Vaseline at 7 d, 14 d, and 21 d post-wounding (Fig. 3).

**Fig. 4:**
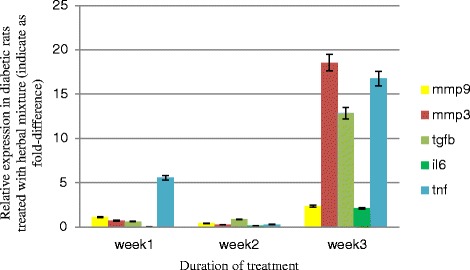
Fold-difference in gene expression in tissue removed from wounds induced in diabetic rats treated with the herbal mixture compared to the Vaseline control at days 7, 14 and 21 of the wound healing process

**Fig. 5:**
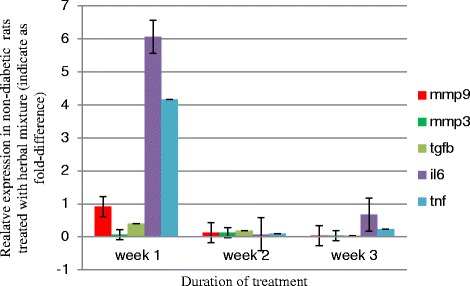
Fold-difference in gene expression in tissue removed from wounds induced in non-diabetic rats treated with the herbal mixture compared with the Vaseline control at days 7, 14 and 21 of the wound healing process

We couldn’t see any significant changes (*p* > 0.05) in the expression of the *Mmp3*, the *Il6*, the *Tnf* α, the *Mmp9* and the *Tgfb1* in diabetic rats treated with herbal mixture in comparison to diabetic rats treated with Vaseline, as for the non-diabetic rats (Figs. 4 and 5).

## Discussion

Here we introduce a herbal mixture that could assist wound healing in diabetic persons. Observational comparisons of wound healing were coupled with gene expression analysis in wound tissue in a time series during healing, and the herbal mixture was compared with control treatment in the type I diabetic compared with control rat subgroups. Physical observation and expression data demonstrate that our herbal formula significantly increases *Mmp3*, *Tgfb1* and *Tnf* α expression in the healing wound over time compared to control treatment with Vaseline. Keratinocytes at a wound site express MMP3 (formerly known as stromelysin 1), which is essential for re-epithelialization and tissue remodeling [2, 39]. MMP3 acts proteolytically on the substrates, fibronectin, laminin, vitronectin, collagens and pro-MMP9, for extracellular matrix remodeling [40, 41]. MMP3 was proposed to be necessary for wound closure, since the rate of wound healing was reported to be reduced in *Mmp3*-deficient mice. The stringing of the MMP3 or the addition of a synthetic inhibitor of the MMP family decelerates wound contraction [42]. MMP3 enhanced endothelial cell proliferation and migration *in vitro* as well as inhibiting apoptosis in these cells*,* suggesting that MMP3 is an indirect regulator of angiogenesis [5]. Here we showed that herbal mixture treatment up-regulated *Mmp3* expression in the diabetic rats compared with non-diabetic rats during the later phase of wound healing. Thus, the ingredients in the herbal mixture are capable of increasing *Mmp3* expression in diabetic rats, which supports a positive effect on extracellular matrix remodeling in the wound even within a diabetic physiological background.

Keratinocyte migration is dependent on MMP9 (formerly known as gelatinase B). In cells with a high glucose concentration, MMP9 expression has been shown to be evaluated in a similar way as has previously been observed in diabetic wounds [11, 43]. Kowluru et al. demonstrated that MMP9 activation under hyperglycemic conditions might activate apoptosis through the caspase 3 pathway [43]. MMP9 accelerates healing of otherwise poorly healing wounds in diabetic individuals, and is implicated in a number of pro-inflammatory events that can induce cytokine activity and potentiate inflammatory response [44]. The mechanism leading to the *Mmp9* up-regulation is poorly understood, but has been associated with increased inflammation. Neutrophils and macrophages permeate the wound during the inflammatory phase, then proceed to phagocytose bacteria and increase *Mmp9* expression [45, 46]. *Mmp9* up-regulation is coordinated by inflammatory cytokines including TNF, IL1B, IL6 and growth factors [43, 47, 48]. However, we did not observe any significant differences in Mmp9 expression at the wound site in diabetic or non-diabetic rats treated with herbal mixture during the 3-week period examined.

Macrophages, T cells, fibroblasts, keratinocytes and endothelial cells can all produce IL6, and contribute particularly to the early inflammatory phase of wound repair [49, 50]. IL6 may regulate leukocyte recruitment to inflammatory sites and fibrotic changes [49]. Wound healing has been demonstrated to also occur in *Il6*-deficient mice [51]. Studies have shown that *Il6* is significantly up-regulated in kidney tissue of diabetic rats [52] and humans [53]. In the present study, *Il6* expression was elevated at day 14 in wound samples from diabetic rats in comparison to non-diabetic rats both treated with the herbal mixture.

TNF is produced by leukocytes and macrophages, whose activation causes acute inflammation via cleavage of TNF from the cell membrane [54, 55]. TNF acts on inflammatory cells, such as monocytes, and is stimulated by higher amount of *MMP9* expression though the activation of the NF-kappa B complex and MAPK signaling [56]. Deactivating TNF signaling in diabetic wounds escalates angiogenesis and wound closure [57]. Apoptosis of fibroblasts in diabetic mice is considerably higher than in non-diabetic mice. Otherwise, TNF appears to induce apoptosis in fibroblasts, keratinocytes and endothelial cells *in vitro* [58, 59]. The migration of fibroblasts and keratinocytes is delayed in diabetics [60]. High TNF levels restrain cell migration. This may occur through elevating SMAD7 levels in the cells, thus, inhibiting signaling activation through SMAD2/3 [57]. In the current study, *Tnf* expression was elevated in wound tissue sampled on days 14 and 21 in diabetic compared with non-diabetic rats all treated with the herbal mixture.

TGFB1 is a multifunctional cytokine released in abundance from platelets, and promotes macrophages, fibroblasts and neutrophils to produce more TGFB1 [16, 61]. TGFB1 is a key mediator implicated in the differentiation and migration of fibroblasts and endothelial cells. Fibroblast migration and proliferation are thought to be important features of wound healing, as fibroblasts are responsible for synthesizing new extracellular matrix proteins, primarily type I and type III collagens [62, 63]. Interestingly, non-healing wounds often show a loss of the TGFB1 signaling [16, 64]. Here we show that *Tgfb1* expression is significantly up-regulated in wound tissue sampled on days 14 and 21 from diabetic rats in comparison to non-diabetic rats all treated with the herbal mixture.

The herbal mixture introduced in this study has not been previously investigated for its effect on wound healing in diabetic rats. However, research on single components, namely *Commiphora molmol*, revealed that the terpenoids (especially furanose squiterpenes) are the active compounds present in myrrh essential oil [33, 65]. Phenolic compounds, alkaloids and saponins have also been detected in extracts of *Commiphora molmol.* Manjula et al. demonstrated *in vitro* that *Commiphora molmol* resin has anti-inflammatory properties, which occur via inhibition of interferon gamma (IFNG), IL-12, TNF, IL1B, and nitric oxide levels [66]. The second component in our mixture is *Aloe Vera,* which possesses substantial amounts of phenol, saponins and anthraquinones, known to be responsible for anti-bacterial, anti-viral and anti-fungal activity [67]. Acemannan is a major carbohydrate fraction obtained from *Aloe Vera* leaves, which produces anti-viral and anti-cancer effects and stimulates the immune system and macrophages [68, 69]. Jettanacheawchankit et al. investigated the effects of Acemannan on the production of keratinocyte growth factor-1 (KGF1),VEGF and type I collagen, and reported that Acemannan is important for oral wound healing [70]. A histological study has revealed that *Aloe vera* enhances wound vascularity, which assists dead tissue removal and increases wound health. The ascorbic acid in *Aloe vera* boosts collagen synthesis to counter-balance collagen breakdown. Collagen is the main extracellular protein involved in granulation tissue homeostasis in a healing wound [1]. Morgan et al. stated that *Aloe Ver*a enhances angiogenesis and wound repair through up-regulating Vegf and *Tgfb1* expression [24]. Vijayalakshmi et al. reported that *Aloe Vera* acts as an anti-inflammatory agent by inhibiting the effect of Mmp9 on peripheral blood mononuclear cells [25]. Abita *et al*. reported that oral administration of *Aloe Vera* improved wound healing and increased the expression of *Vegf* and *Tgfb1*in type 2 diabetic rats [26]. The leaves of the henna plant contain phytochemical ingredients such as tannin, gallic acid, glucose, mannitol, fat, resin, flavonoids, coumarins and anthraquinones [71, 72]. Habbal *et al*. showed that henna leaf extracts are efficient in preventing infections by inhibiting the growth of microorganisms [73]. Nilforoushzadeh et al. evaluated the effects of *Adiantum capillus-veneris* on wound healing, and reported that this plant promoted angiogenic effects and proliferation of endothelial cells in vitro [36]*.* The antioxidant activity of *Adiantum capillus-veneris* could be the result of polyphenolic and flavonoid activity [74]. Flavonoids are recognized for their antioxidant, anti-inflammatory, and cell protective properties [75, 76].

## Conclusions

The present study demonstrated that the prescribed herbal mixture is effective for wound healing and improves conditions at the wound site to promote better closure and healing.

## Abbreviations

ECM: Extracellular matrix
GAPDH: Glyceraldehyde 3-phosphate dehydrogenase
IL-6: Interleukin-6
MMPs: Matrix metalloproteases
PCR: Polymerase chain reaction
TGFβ1: Transforming growth factor beta1
TNFα: Tumor necrosis factor α
